# Deficits in hippocampal-dependent memory across different rodent models of early life stress: systematic review and meta-analysis

**DOI:** 10.1038/s41398-021-01352-4

**Published:** 2021-04-20

**Authors:** Mariana Rocha, Daniel Wang, Victor Avila-Quintero, Michael H. Bloch, Arie Kaffman

**Affiliations:** 1 Department of Psychiatry, Yale University School of Medicine, 300 George Street, Suite 901, New Haven, CT 06511 USA; 2 Child Study Center, Yale University School of Medicine, 230 South Frontage Road, New Haven, CT 06519 USA

**Keywords:** Learning and memory, Pathogenesis, Scientific community

## Abstract

Exposure to early life stress (ELS) causes abnormal hippocampal development and functional deficits in rodents and humans, but no meta-analysis has been used yet to quantify the effects of different rodent models of ELS on hippocampal-dependent memory. We searched PubMed and Web of Science for publications that assessed the effects of handling, maternal separation (MS), and limited bedding and nesting (LBN) on performance in the Morris water maze (MWM), novel object recognition (NOR), and contextual fear conditioning (CFC). Forty-five studies met inclusion criteria (*n* = 451–763 rodents per test) and were used to calculate standardized mean differences (Hedge’s *g*) and to assess heterogeneity, publication bias, and the moderating effects of sex and species (rats vs. mice). We found significantly lower heterogeneity in LBN compared to handling and MS with no consistent effects of sex or species across the three paradigms. LBN and MS caused similar cognitive deficits in tasks that rely heavily on the dorsal hippocampus, such as MWM and NOR, and were significantly different compared to the improved performance seen in rodents exposed to handling. In the CFC task, which relies more on the ventral hippocampus, all three paradigms showed reduced freezing with moderate effect sizes that were not statistically different. These findings demonstrate the utility of using meta-analysis to quantify outcomes in a large number of inconsistent preclinical studies and highlight the need to further investigate the possibility that handling causes different alterations in the dorsal hippocampus but similar outcomes in the ventral hippocampus when compared to MS and LBN.

## Introduction

Early life stress (ELS) impairs the development of several interconnected brain regions including the hippocampus, prefrontal cortex, striatum, and amygdala^1,2^. Of these, reduced hippocampal volume and abnormal hippocampal function are some of the most reproducible findings in individuals exposed to ELS^1,3,4^. This is not surprising given that the hippocampus undergoes substantial developmental changes during childhood that are highly sensitive to stress^5–7^. The hippocampus also forms extensive connections with other brain regions involved in declarative memory, spatial learning, threat detection, stress reactivity, and mood regulation^8,9^. These features make the hippocampus a potentially important hub in mediating several of the cognitive, emotional, and physiological abnormalities seen in individuals exposed to ELS^1,3,4^.

Elucidating the mechanisms by which ELS affects hippocampal development is a challenging task in humans because of the inherent complexity and heterogeneity of the adversities, genetic variability, and numerous additional variables that are difficult to control in clinical settings^10^. In addition, causally linking structural and functional changes in the hippocampus with alterations in anxiety, stress reactivity, or cognition are practically impossible to do in humans. Nevertheless, the conserved nature by which the hippocampus develops in mammalian species^5,11^ and the observation that rodent models of ELS cause significant impairment in its development and long-term function^10^ suggest that work in rodents may clarify important details about the role that abnormal hippocampus development plays in the long-term consequences of ELS. Indeed, an elegant body of work has shown that chronically elevated levels of the neuropeptide corticotrophin-releasing hormone (CRH) mediate the synaptic and hippocampal-dependent memory deficits in rodents exposed to a rodent model of ELS known as limited bedding and nesting (LBN)^12^.

Several key questions regarding the effects of ELS on hippocampal function in rodents remain unresolved. For example, it is currently unclear whether some types of ELS lead to more severe hippocampal-dependent deficits, whether males and females are equally sensitive, and if some tests are more sensitive for detecting hippocampal-dependent cognitive deficits caused by ELS. Even within the same ELS paradigm, different research groups report variable outcomes and the literature is replete with examples of inconsistent findings^10,13,14^. These inconsistencies appear to be related to many factors such as different genetic backgrounds, poor standardization of procedures across labs, and other stochastic variables^10,14–16^. To address these issues, we conducted a systematic review and subsequent meta-analysis examining the effects of three types of ELS: handling (also known as brief maternal separation), maternal separation (MS), and LBN on hippocampal function in rodents (mice and rats). Hippocampal-dependent memory was assessed using 3 behavioral tests: (1) the Morris water maze (MWM), (2) Novel Object Recognition (NOR), and (3) Contextual Fear Conditioning (CFC). We chose these tests because they all require normal hippocampal function^8,17,18^ and are commonly tested in rodents exposed to ELS^13,14^. Moreover, the MWM and the NOR rely more on the dorsal hippocampus^8,19–21^, whereas freezing behavior in the CFC is thought to have a strong ventral hippocampus component^8,22–25^. Nevertheless, performance in these tests, especially steps that involve consolidation and retrieval, also require the prefrontal cortex and other cortical regions that need to be considered when interpreting behavioral outcomes in these tests^17,18,26–28^.

Although two systematic reviews have previously examined the effects of different types of ELS on behavioral outcomes in rodents, including spatial learning^13,14^ they were qualitative in nature and did not include a meta-analysis. Meta-analysis is routinely used in clinical settings but has rarely been used to address conflicting results in preclinical studies in rodents^29^. In fact, we are aware of only two meta-analyses examining the effects of ELS on behavioral outcome in rodents, one assessing pain sensitivity^30^ and the other examining anxiety-like-behavior^31^. No meta-analysis to date has assessed the effects of ELS on spatial learning and none of the available meta-analysis included the LBN which is one of the most commonly used ELS paradigms in rodents^32^.

The primary goals of this meta-analysis were to compare the effect sizes and heterogeneity of the three different ELS paradigms in the MWM, NOR, and CFC. We also examined the effects of several moderators such as sex, species (i.e., mice vs. rats), separation index (i.e., the number of days pups were separation X length of the separation), and temperature of isolation on cognitive outcomes. Lastly, we assessed for possible publication bias and its impact on behavioral outcomes in these tests.

## Methods

### Search strategy

Two reviewers (MR, DW) searched the electronic databases of PubMed and Web of Science on September 30th, 2019 for relevant studies using the following searches: (early life stress OR ELS OR postnatal stress OR maternal separation OR neonatal stress OR limited bedding and nesting OR LBN OR brief maternal separation OR BMS OR handling) AND (mice OR mouse OR mus musculus OR rats OR rat) AND (Morris water maze OR MWM OR novel object recognition OR NOR OR novel object location OR NOL OR contextual fear conditioning OR CFC). Studies were limited to English language articles. Additional citations came from previous systematic reviews^13,14,32,33^. Studies obtained from the search, the titles, and abstracts were examined by the two reviewers (MR, DW) to determine preliminary inclusion. Discrepancies were addressed by the reviewers through discussion and through conversation with senior reviewers (AK, MHB). Despite a concerted effort to identify all relevant studies, it is possible that we have missed some studies due to the enormity of the literature and the different names used to describe these paradigms. In addition, we did not register this study with PROSPERO prior to extracting the data and therefore are not able to do this at this point. However, we are confident that no previous meta-analyses have examined this question before.

### Study selection and data extraction

After preliminary inclusion, studies were carefully read to determine if inclusion criteria were met. See Supplemental Information for additional details regarding selection criteria and data extraction are available in the supplementary information.

### Data analysis

Statistical Analyses were conducted using the Comprehensive Meta-Analysis Version 3.0 software (Biostat, 2016). Outcomes of interest were measures of hippocampal-dependent Memory in the MWM, NOR, and CFC. Details regarding specific behavioral measurements are available in the supplementary information. Hedge’s g was used as the pooled measurement of effect size as it is preferred over Cohen’s d for small samples, which are common in animal studies^34^. Both fixed and random effects were calculated and are presented in the results section. Random effect analysis was used as the primary analysis to account for the large heterogeneity between studies. Fixed-effect analysis was conducted as a sensitivity analysis because random-effects models may be overly conservative in situations where a relatively small number of studies is available. Publication bias was assessed using funnel plots and the Egger’s test, and heterogeneity was measured utilizing the *I*^2^ statistics and Chi-square test for heterogeneity^35,36^. Details regarding the analyses for moderating effects of sex, species, separation index and temperature are available in the supplemental information.

## Results

### Selection studies

Figure S1 depicts the selection strategy for included studies. In total, 1435 articles were identified for consideration, of which 45 studies were eligible for inclusion. Reasons for exclusion of studies are identified in the Supplemental Information Fig. S1. Table 1 describes the characteristics of our included studies and additional details are available in the supplemental information Tables S1 and S2.

**Table 1:** Alphabetical list of studies included in the meta-analysis.

Reference	ELS paradigm	Species-strain	Sex	Sample size	Outcome(s) tested
Aisa, 2007^45^	MS	Rat-Wistar	M	10–12/group	MWM, NOR
Asia, 2008^46^	MS	Rat-Wistar	F	10/group	NOR
Banqueri, 2018^47^	MS	Rat-Wistar	F	10/group	MWM
Baudin, 2012^48^	MS	Rat-LH	M	12/group	MWM
Burnson, 2005^49^	LBN	Rat-SD	M	8–11/group	MWM, NOR
Cao, 2014^50^	MS	Rat-SD	M	9–10/group	MWM
Chocyk, 2014^51^	MS	Rat-Wistar	M and F	20/group	CFC
Couto-Pereira, 2019^52^	MS, Handling	Rat-Wistar	M	13/group	CFC
Cui, 2006^53^	LBN	Rat-SD	M	8/group	MWM
Dalle, 2017^54^	MS	Rat-SD	M	10–20/group	MWM
Diehl, 2012^55^	MS	Rat-Wistar	M	9/group	MWM
Diehl, 2014^56^	MS	Rat-Wistar	M and F	8/group	CFC
Fegnolio, 2005^40^	Handling	Rat-SD	M	8–11/group	MWM, NOR
Guijarro, 2007^57^	MS, Handling	Rat-Wistar	M	14–24/group	CFC
Hoeijmakers, 2018^58^	LBN	Mouse-C57B1/6J	M	9–12/group	MWM, NOR
Huang, 2002^59^	MS	Rat-SD	M	8–9/group	MWM
Ivy, 2010^60^	LBN	Rat-SD	M	13–23/group	MWM, NOR
Kanatsou, 2017^61^	LBN	Mouse, C57BI6, C57BL/6N	M	10/group	CFC
Kosten, 2006^62^	MS, Handling	Rat-SD	M and F	6–8/group	CFC
Lai, 2006^63^	MS	Rat-SD	M	20–22	MWM
Li, 2018^64^	Handling	Mouse-BALB/cCrSlc	M	8–10/group	MWM
Manzano-Nieves, 2018^65^	LBN	Mouse-C57BL/6N	M and F	7–14/group	CFC
Molet, 2016^66^	LBN	Rat-SD	M	6/group	NOR
Naninik, 2015^67^	LBN	Mouse-C57B1/6J	M and F	6/group	MWM, NOR
Naninik, 2017^68^	LBN	Mouse-C5Bl/6J	M	13–14/group	MWM, NOR
Noschang, 2010^69^	Handling	Rat-Wistar	M and F	7–10/group	MWM
Plescia, 2014^70^	Handling	Rat-Wistar	F	16–26/group	MWM, NOR
Pusceddu, 2015^71^	MS	Rat-SD	F	10/group	NOR
Reshetnikov, 2018^72^	MS, Handling	Mouse-c57B1/6	M	8–10/group	NOR
Rice, 2008^73^	LBN	Mouse-C57BL/6J	M	6–13/group	MWM, NOR
Solas, 2010^74^	MS	Rat-Wistar	M	15/group	MWM, NOR
Sun, 2014^75^	MS	Rat-Wistar	M and F	7–13/group	MWM, CFC
Uysal, 2005^76^	MS	Rat-Wistar	M and F	8/group	MWM
Wang L., 2011^77^	MS	Mouse-BALB/cJ	F	16/group	NOR
Wang XD Rammes, 2011^78^	LBN	Mouse, 129S2/Sv X C57BL/6J	M	18–20/group	MWM
Xiong, 2014^79^	MS	Rat-SD	F	8/group	CFC
Xiong, 2015^80^	MS	Rat-SD	M	12–16/group	MWM, CFC
Xu, 2018^81^	MS	Rat-SD	M and F	8/group	MWM
Xue, 2013^82^	MS	Rat-Wistar	M	6/group	MWM
Zaharia, 1996^83^	Handling	Mouse, BALB/cByJ, C57BL/6ByJ	M	16–18/group	MWM
Zhang, 2014^84^	MS	Rat-SD	M	11–13/group	MWM
Zoicas, 2016^85^	MS	Mouse-CD1	M	12/group	NOR

*LH* Long-Havens, *M* Males, *F* Females, *SD* Sprague-Dawley.

For additional details see Tables S1 and S2 in the Supplemental Information.

### Morris water maze (MWM): latency to find platform training

**Fig. 1:**
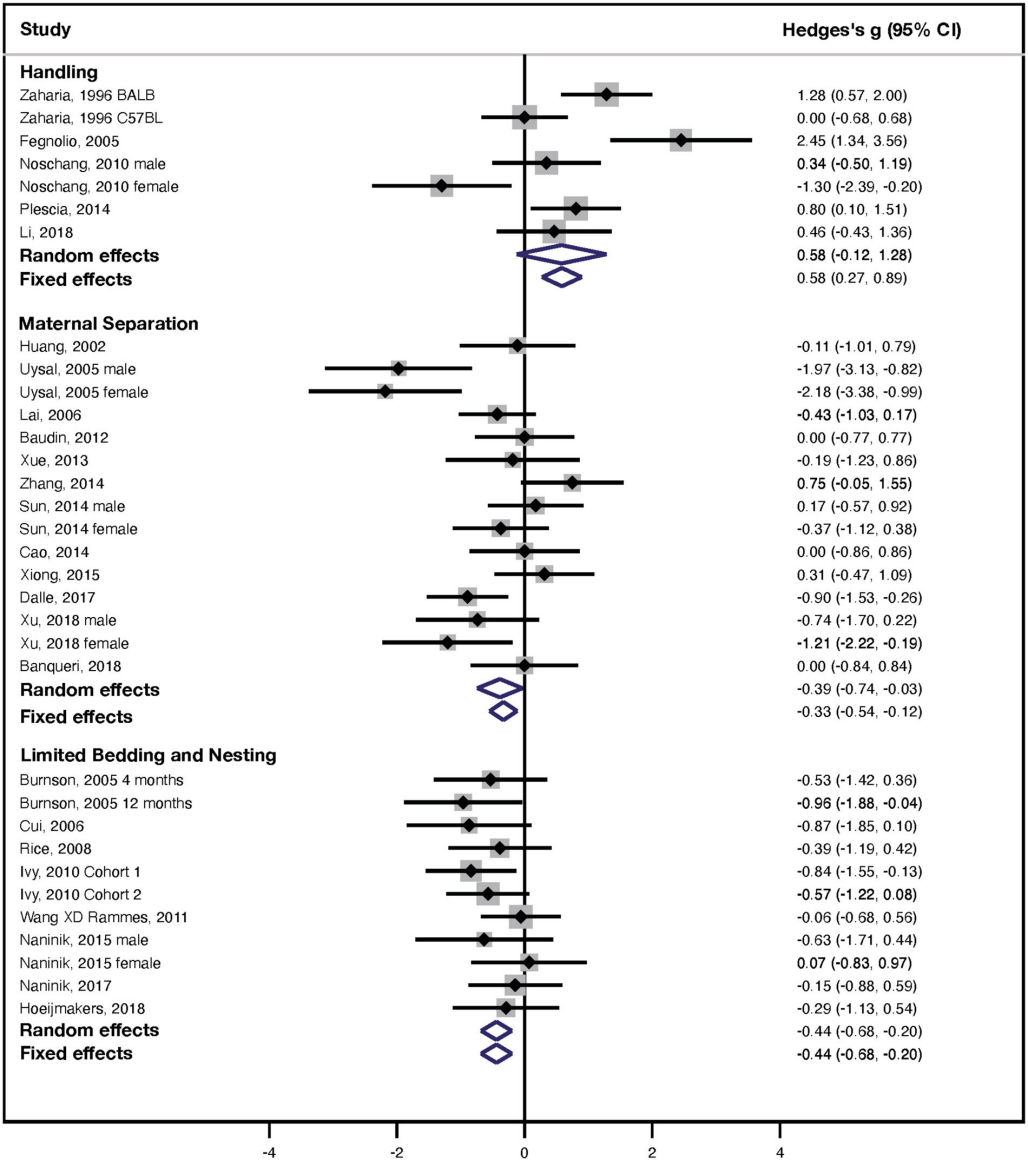
Latency to find platform-MWM. Forest plot depicting the effects of different rodent models of ELS on latency to find a platform in the MWM task. Studies are organized based on ELS paradigms with handling at the top, followed by MS in the middle, and LBN at the bottom. Hedges *g* effect sizes for individual studies are shown graphically in the middle column with numerical summaries in the right column. Summaries of random and fixed effect sizes are shown as blue diamonds at the bottom of each ELS paradigm.

A forest plot summary of the effects of handling (7 studies, *n* = 162 rodents), MS (15 studies, *n* = 338 rodents), and LBN (11 studies, *n* = 243 rodents) on spatial learning in the MWM is shown in Fig. 1 and Fig. S2. Test for subgroup differences demonstrated significant differences in effect size (Hedge’s *g*) between the three experimental paradigms (random effect: *χ*^2^ = 7.34, df = 2, *p* = 0.03; fixed effect: *χ*^2^ = 29.3, df = 2, *p* < 0.0005). Post-hoc pairwise comparisons found significant differences in effect sizes between handling and MS (random effect: *χ*^2^ = 5.81 df = 1, *p* = 0.02; fixed effect: *χ*^2^ = 22.9 df = 1, *p* =< 0.0005) and between handling and LBN (random effect: *χ*^2^ = 7.3, df = 1, *p* = 0.01; fixed effect: *χ*^2^ = 26.3, df = 1, *p* < 0.0005) but not between MS and LBN (random effect: *χ*^2^ = 0.06 df = 1, *p* = 0.81; fixed effect: *χ*^2^ = 0.44 df = 1, *p* = 0.51).

Exposure to handling reduced the latency to find the platform (Fig. 1, top and Fig. S2), an outcome that was not significant for random effect (Hedge’s *g* = 0.58 ± 0.36, 95% Confidence Interval (CI) = −0.12–(+1.28), *z* = 1.62, *p* = 0.11, *k* = 7) but significant using fixed-effect analysis (Hedge’s *g* = 0.58 ± 0.16, 95% Confidence Interval (CI) = 0.27–0.89, *z* = 3.70, *p* = 0.0002, *k* = 7). There was significant heterogeneity between studies (*I*^2^ = 80%, *Q* = 30, df = 6, *p* < 0.0005) but no evidence for publication bias based on funnel plot asymmetry or Egger’s test for handling (Supplemental information Fig. S3A), Egger’s test *p* = 0.97). Sex (*Q* = 1.45, df = 1, *p* = 0.22) or species (i.e., rats vs. mice, *Q* = 0.00, df = 1, *p* = 0.99) did not affect latency to find the platform.

In contrast to the reduced latency seen in handling, exposure to MS was associated with a significant increase in the latency to find the platform (random effect: Hedge’s *g* = −0.39 ± 0.18, 95% CI = −0.74–(−0.03), *z* = −2.14, *p* = 0.032, *k* = 15; fixed effect: Hedge’s *g* = −0.30 ± 0.11, 95% CI = −0.52–(−0.09), *z* = −2.75, *p* = 0.006, *k* = 15, Fig. 1, middle). There was significant heterogeneity between studies (*I*^2^ = 62%, Q = 37, df = 14, *p* = 0.0007) with no evidence of publication bias based on the funnel plot (Supplemental information Fig. S3B) or the Egger’s test (*p* = 0.14). Stratified subgroup analysis demonstrated no significant effect of sex (*Q* = 1.9, df = 1, *p* = 0.17), and all MS studies were done in rats, preventing us from assessing the effect of species on latency to find the platform. Moderator analyses found no significant effect of separation index (*β* = −0.0063 ± 0.0063, 95% CI = −0.019-(0.062), *Z* = −0.99, *p* = 0.32, *k* = 15), but significant effect of separation temp (*β* = −0.44 ± 0.18, 95% CI = −0.8–(−0.079), *Z* = −2.39, *p* = 0.017, *k* = 15) with longer latency to find the platform associated with incubation at a higher temperature during the MS procedure.

Similar to outcomes seen in MS, rodents exposed to LBN showed a significant increase in latency to find the platform (random and fixed effects: Hedge’s *g* = −0.44 ± 0.12, 95% CI = −0.68–(−0.20), *z* = −3.59, *p* < 0.0005, k = 11; Fig. 1, bottom). The heterogeneity between LBN studies was minimal and non-significant (*I*^2^ = 0%, *Q* = 6.9, df = 10, *p* = 0.73). There was no evidence for publication bias based on funnel plot symmetry and a non-significant Egger’s test (*p* = 0.35, Supplemental information Fig. S3C). No significant effect of sex was seen in LBN studies (*Q* = 1.32, df = 1, *p* = 0.25), but there was a significant effect of species (*Q* = 4.66, df = 1, *p* = 0.033). Further examination revealed that LBN increased latency to find the platform in rats (Hedge’s *g* = −0.73 ± 0.18, 95% CI = −1.1–(−0.4), *z* = −4.02, *p* < 0.0005, *k* = 5), but not in mice (Hedge’s *g* = −0.20 ± 0.17, 95% CI = −0.53–(+0.12), *z* = −1.2, *p* = 0.23, *k* = 6).

### MWM: probe trial

**Fig. 2:**
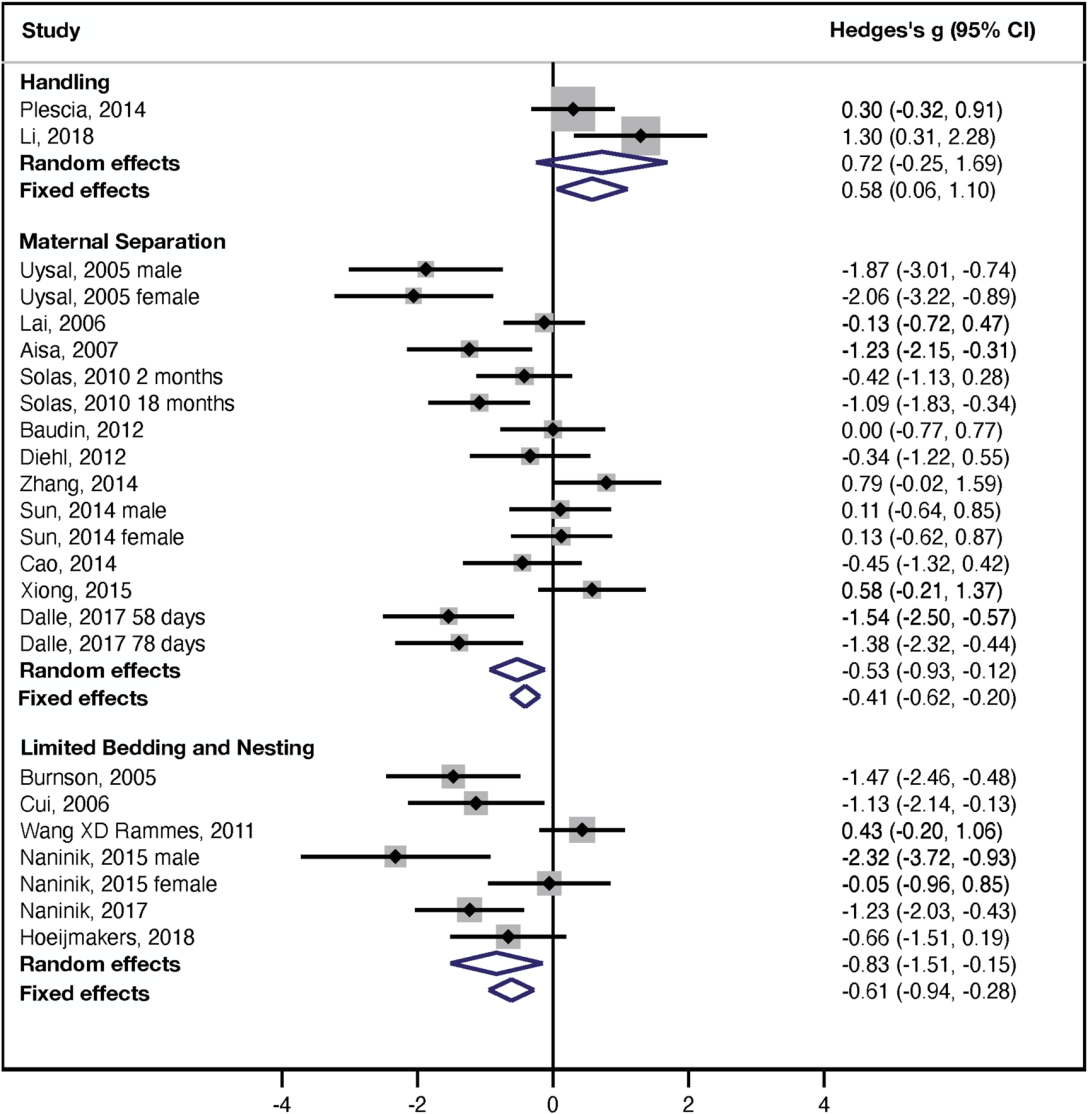
Probe trial, MWM. Forest plot for the effects of handling, MS, and LBN on performance in the probe trial of the MWM. Studies are organized based on ELS paradigms with the effect size for individual studies shown graphically in the middle column and numerical summaries available in the right column. Overall random and fixed effect sizes are shown as blue diamonds at the bottom of each ELS paradigm.

The forest plot summary for the effects of handling (2 studies, *n* = 60 rodents), MS (15 studies, *n* = 343 rodents), and LBN (7 studies, *n* = 162 rodents) on performance in the probe trial of the MWM are shown in Fig. 2. Rodents exposed to MS and LBN spent significantly less time swimming in the correct quadrant whereas handling was associated with a non-significantly increased time spent in the correct quadrant compared to controls. Tests for subgroup differences demonstrated significant differences in overall effect sizes between the three experimental paradigms (random effect: *χ*^2^ = 6.99, df = 2, *p* = 0.03; fixed effect: *χ*^2^ = 14.9, df = 2, *p* = 0.001) that was due to increased time swimming in the correct quadrant in handling compare to MS (random effect: *χ*^2^ = 5.45, df = 1, *p* = 0.02; fixed effect: *χ*^2^ = 11.9, df = 1, *p* = 0.0006) and LBN (random effect: *χ*^2^ = 6.63 df = 1, *p* = 0.01; fixed effect: *χ*^2^ = 14.3, df = 1, *p* < 0.0005). Comparison between MS and LBN was not significant using either the random-effects (*χ*^2^ = 0.57, df = 1, *p* = 0.45) or fixed-effects models (*χ*^2^ = 1.02 df = 1, *p* = 0.31).

Since only two handling studies were included (Table S2), the outcomes for the probe trial should be considered exploratory and did not include analyses of publication bias or on the effects of sex and species. Nevertheless, handling increased time swimming in the correct quadrant (Fig. 2, top), an outcome that was not significant using random effect (Hedge’s *g* = 0.72 ± 0.49, 95% CI = −0.25–(+1.69), *z* = 1.5, *p* = 0.14, *k* = 2) but significant using a fixed-effect analysis (Hedge’s *g* = 0.58 ± 0.27, 95% CI = 0.06–1.10, *z* = 2.2, *p* = 0.029, *k* = 2). The heterogeneity between the studies was large but did not reach statistical significance given the small number of studies (*I*^2^ = 65%, *Q* = 3, df = 1, *p* = 0.091).

**Fig. 3:**
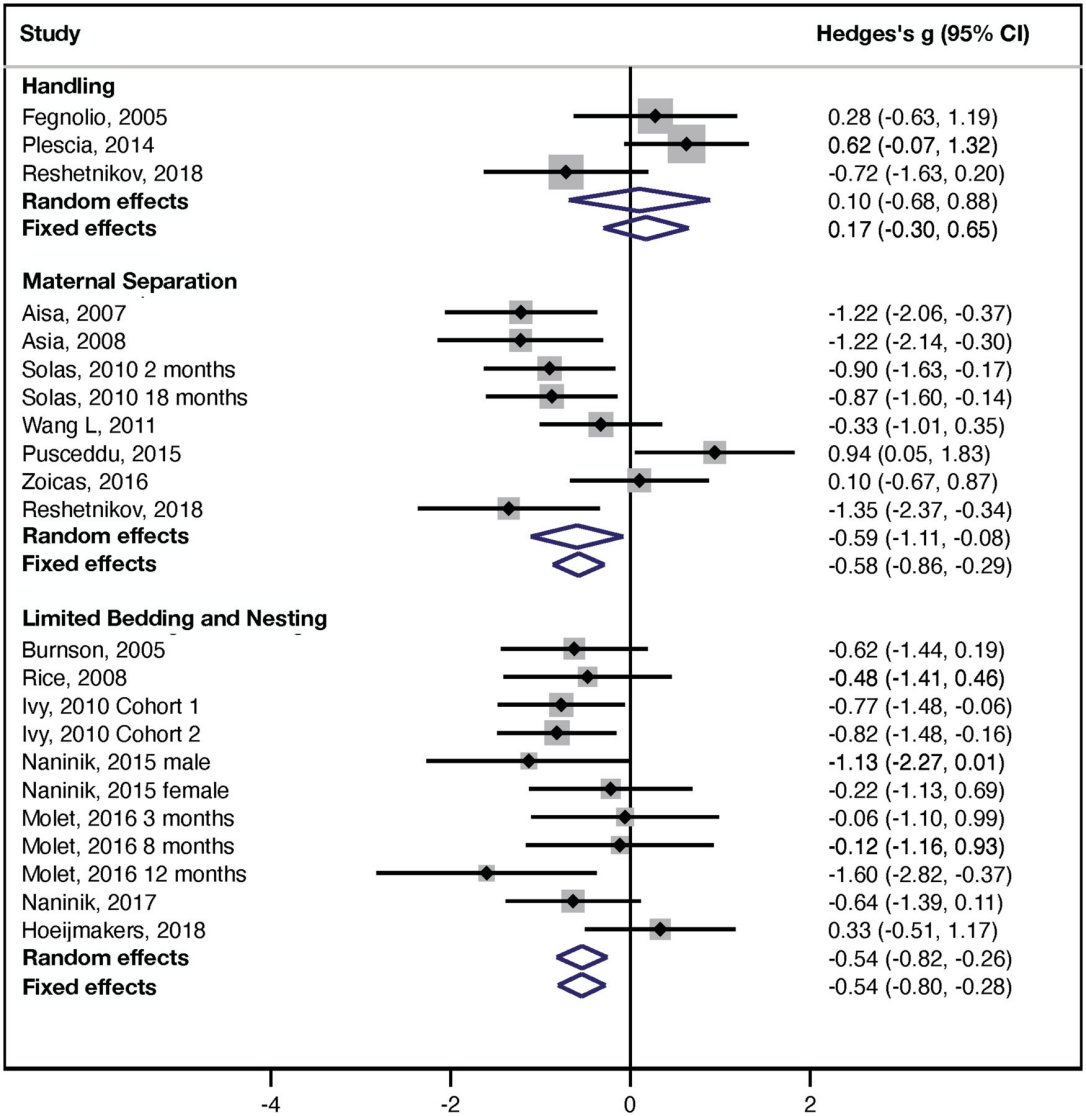
Forest plot-NOR. Forest plot examining the effects of handling, MS, and LBN on NOR test. Studies are organized based on ELS paradigms. Hedges g effect sizes for individual studies are shown graphically in the middle column with numerical summaries provided in the right column. Summaries of random and fixed effect sizes are shown as blue diamonds at the bottom for each ELS paradigm.

Exposure to MS reduced time swimming in the correct quadrant (random effect: Hedge’s *g* = −0.53 ± 0.21, 95% CI = −0.93–(−0.12), *z* = −2.6, *p* = 0.01, *k* = 15; fixed effect: Hedge’s *g* = −0.41 ± 0.11, 95% CI = −0.62–(−0.20), *z* = −3.8, *p* = 0.00014, *k* = 15, Fig. 3 middle). The heterogeneity between the studies was large and statistically significant (*I*^2^ = 72%, *Q* = 50, df = 14, *p* < 0.0005). Funnel plot asymmetry suggested a possible publication bias (Supplemental information Fig. S4A), a finding that was confirmed using the Egger’s test (*p* = 0.009). Nevertheless, the effect of MS remained significant after adjusting for funnel plot asymmetry using Duval and Tweedie’s Trim-and-Fill method, Hedge’s *g* = −0.30, 95% CI = −05–(−0.09). There was no significant effect of sex (*Q* = 0.24 df = 1, *p* = 0.62) and all MS studies were conducted in rats (Table S2), preventing us from assessing the effect of species on performance in the probe trial. Moderator analyses found no significant effect of separation index (*β* = −0.0032 ± 0.0063, 95% CI = −0.016–(−0.0092), *Z* = −0.51, *p* = 0.61, *k* = 14). However, as with the latency to find the platform there was a significant effect of separation temp on outcomes in the probe trial with worse performance in rodents exposed to higher temp during the MS procedure (*β* = −0.33 ± 0.12, 95% CI = −0.56–(−0.10), *Z* = −2.80, *p* = 0.05, *k* = 8).

Similar to MS, rodents exposed to LBN showed significantly reduced time swimming in the correct quadrant (Fig. 2 bottom). This outcome had a large effect size that was highly significant (random effect: Hedge’s g = −0.82 ± 0.35, 95% CI = −2.40–(−0.51), *z* = −2.4, *p* = 0.02, *k* = 7; fixed effect: Hedge’s *g* = −0.61 ± 0.17, 95% CI = −0.94–(−0.28), *z* = −3.6, *p* < 0.0005, *k* = 7). The heterogeneity within the LBN studies was large and statistically significant (*I*^2^ = 75%, *Q* = 10, df = 6, *p* = 0.001). There was evidence of significant publication bias for LBN studies (Supplemental information Fig. S4B, and egger’s test: *p* = 0.02). Using the trim-and-fill method to adjust for publication bias, findings remained significant in a fixed-effect (Hedge’s *g* = −0.52,95% CI = −0.85–(−0.20), but not a random-effect model (Hedge’s *g* = −0.65, 95% CI = −1.33–0.02). Meta-regression demonstrated no significant effect of sex (*Q* = 2.3, df = 1, *p* = 0.13) or species (*Q* = 1.3, df = 1, *p* = 0.25).

### Novel object recognition (NOR)

A forest plot summarizing the effects of handling (3 studies, *n* = 67 rodents), MS (8 studies, *n* = 197 rodents), and LBN (11 studies, *n* = 232 rodents) on performance in the NOR test is shown in Fig. 3. Tests for subgroup differences found no significant differences in effect size using random effect (*χ*^2^ = 2.45, df = 2, *p* = 0.29), but significant differences using fixed-effect analysis (*χ*^2^ = 7.94, df = 2, *p* = 0.019). However, the relationship between the three paradigms was similar to outcomes in the latency to find platform and probe trial (Fig. 3).

Handling had a small effect size that was not significant (random effect: Hedges *g* = 0.10 ± 0.40, 95% CI = −0.68–(+0.88), *z* = 0.25, *p* = 0.80, *k* = 3; fixed effect: Hedges *g* = 0.17 ± 0.24, 95% CI = −0.30–(+0.65), *z* = 0.72, *p* = 0.47, *k* = 3, Fig. 3, top). There was substantial heterogeneity between studies that did not reach statistical significance (*I*^2^ = 62%, *Q* = 5, df = 2, *p* = 0.071), most likely due to the small number of available studies (Table S2). The small number of studies also prevented us from assessing publication bias or the effects of sex and species on performance in the NOR test.

Rodents exposed to MS showed impaired memory in the NOR test that was moderate in size and statistically significant (random effect: Hedges *g* = −0.59 ± 0.26, 95% CI = −1.1–(−0.08), *z* = −2.27, *p* = 0.023, *k* = 8; fixed effect: Hedges *g* = −0.58 ± 0.14, 95% CI = −0.86–(−0.29), z = −3.97, *p* < 0.0005, *k* = 8, Fig. 3, middle). There was substantial heterogeneity between studies (*I*^2^ = 69%, *Q* = 22, df = 7, *p* = 0.0022) but no evidence of publication bias based on funnel plot asymmetry (Supplemental information Fig. S5A) or the Egger’s test (*p* = 0.69). There was no significant effect of species (*Q* = 0.15, df = 1, *p* = 0.70) or sex: (Q = 0.95, df = 1, *p* = 0.33). Moderator analyses found a significant effect of separation index on performance in the NOR, with longer separation associated with worse outcomes (*β* = −0.047 ± 0.017, 95% CI = −0.080–(−0.013), *Z* = −2.73, *p* = 0.0064, *k* = 8). No analysis was done for separation temperature because only 4 studies reported it (Table S1).

Exposure to LBN caused a significant impairment in the NOR test (random effect: Hedges *g* = −0.54 ± 0.14, 95% CI = −0.82–(−0.26), *z* = −3.79, *p* < 0.0005, *k* = 11; fixed effect: Hedges *g* = −0.54 ± 0.13, 95% CI = −0.80–(−0.28), *z* = −4.06, *p* < 0.0005, *k* = 11, Fig. 3, bottom). Heterogeneity within the LBN studies was small and non-significant (*I*^2^ = 11%, *Q* = 11, df = 10, *p* = 0.34). There was no evidence of publication bias based on funnel plot asymmetry (Supplemental information Fig. S5B) or the Egger’s test (*p* = 0.96) and no significant effect species (*Q* = 1.05, df = 1, *p* = 0.31). Only one study was conducted in females, preventing us from assessing the effect of sex (Table S2).

### Contextual fear conditioning (CFC)

**Fig. 4:**
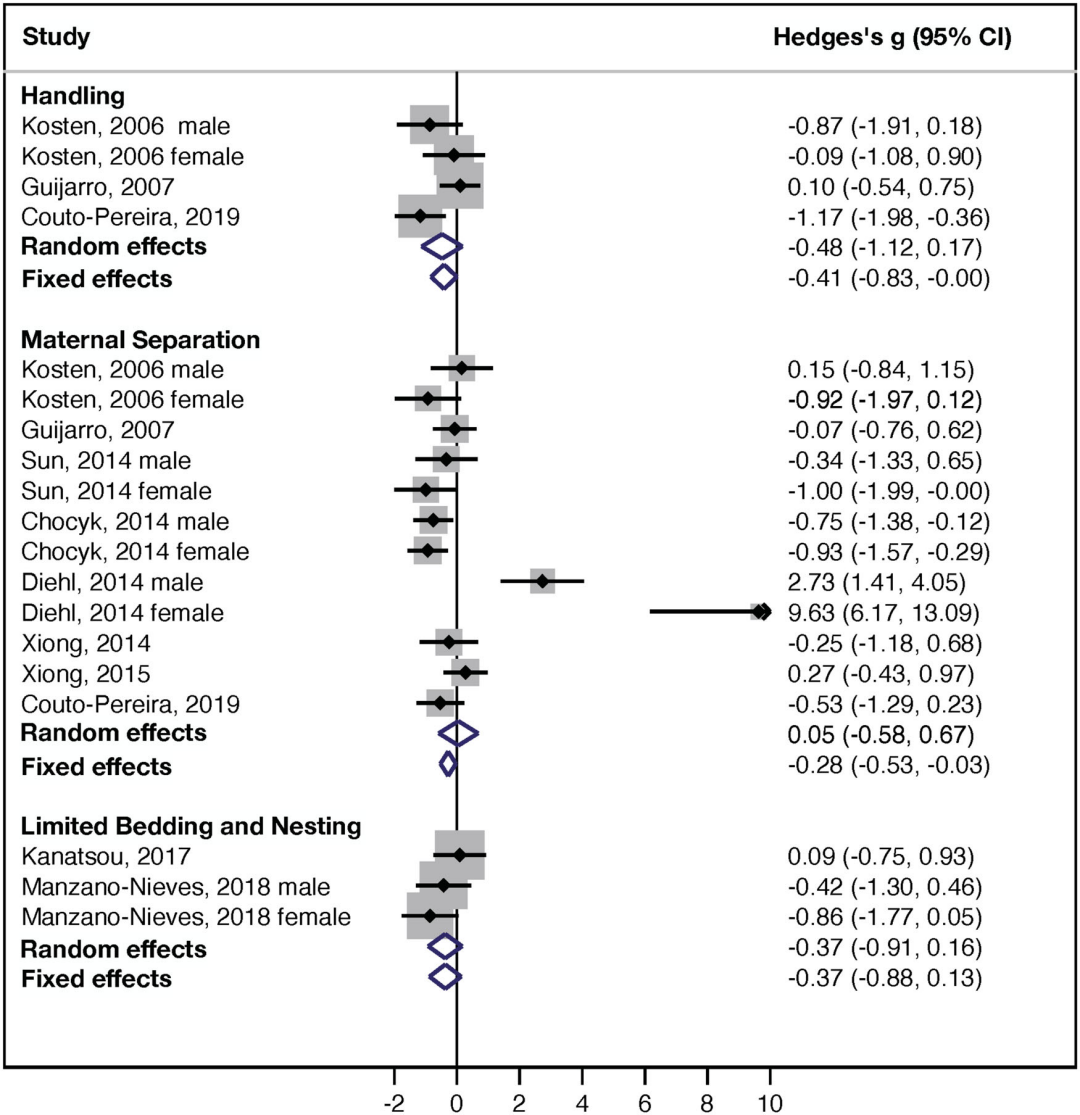
Forest plot-CFC. Forest plot for the effects of handling, MS and LBN on freezing behavior in the CFC test. Studies are organized based on ELS paradigms with the effect size for individual studies shown graphically in the middle column and numerical summaries available in the right column. Summaries of random and fixed effect sizes are shown as blue diamonds at the bottom of each ELS paradigm.

A forest plot summary for the overall effect sizes of handling (4 studies, *n* = 92 rodents), MS (12 studies, *n* = 299 rodents), and LBN (3 studies, *n* = 60 rodents) on freezing behavior in the CFC are shown in Fig. 4. Unlike outcomes in the MWM and NOR, tests for subgroup differences did not reveal significant differences in effect size (Hedge’s *g*) between the three experimental paradigms (random effect: *χ*^2^ = 1.52, df = 2, *p* = 0.4; fixed effect: *χ*^2^ = 0.34, df = 2, *p* = 0.84, Fig. 4).

In contrast to the improved cognition in the MWM and the lack of impact in the NOR, exposure to handling reduced contextual freezing suggesting impaired hippocampal-dependent memory. The effect size was moderate and did not reach statistical significance using random-effect analysis, most likely due to the small number of available studies (Hedge’s *g* = −0.48 ± 0.33, 95% CI = −1.12–(+0.17), *z* = −1.44, *p* = 0.15, *k* = 4). The effect of handling on CFC was significant when the fixed-effect analysis was performed (Hedge’s *g* = −0.41 ± 0.21, 95% CI = −0.83–(−0.001), *z* = −1.97, *p* = 0.049, *k* = 4, Fig. 4 top). There was a large heterogeneity that did not reach statistical significance (*I*^2^ = 57%, *Q* = 6.92, df = 3, *p* = 0.075) and too few studies were available to assess publication bias or the effects of sex and species.

MS also reduced contextual freezing, an effect that was not significant for random effect (Hedges *g* = 0.05 ± 0.32, 95% CI = −0.58–(+0.67), z = 0.15, *p* = 0.88, *k* = 12), but significant for fixed-effect analysis (Hedges *g* = −0.28 ± 0.13, 95% CI = −0.53–(−0.03), z = −2.24, *p* = 0.025, *k* = 12, Fig. 4, middle). As with other studies involving MS, there was large and highly significant heterogeneity between studies (*I*^2^ = 83%, *Q* = 64.8, df = 11, *p* < 0.0005). There was also substantial evidence for publication bias based on funnel plot asymmetry and the Egger’s test (*p* = 0.006) that were highly influenced by 1 outlier study (46) (Supplemental information Fig. S6). We were unable to assess the effect of species because all studies were conducted in rats (Table S2) and there was no significant effect of sex (*Q* = 0.08, df = 1, *p* = 0.78). Moderator analyses of MS studies found no significant effect of separation index (*β* = −0.015 ± 0.0098, 95% CI = −0.035–(0.041), *Z* = −1.55, *p* = 0.12, *k* = 12) or separation temp (*β* = 0.063 ± 0.087, 95% CI = −0.11–(0.23), Z = 0.72, *p* = 0.47, *k* = 10).

Rodents exposed to LBN also showed reduced contextual freezing, with a moderate effect size that did not reach significance most likely due to the small number of available studies (random effect: Hedge’s *g* = −0.37 ± 0.27, 95% CI = −0.91 –(+0.16), z = −1.36, *p* = 0.17, *k* = 3; fixed effect: Hedge’s *g* = −0.37 ± 0.26, 95% CI = −0.88–(+0.13), *z* = −1.44, *p* = 0.15, *k* = 3, Fig. 4 bottom). Heterogeneity between studies was small and did not reach statistical significance (*I*^2^ = 12%, *Q* = 2.27, df = 2, *p* = 0.32) and too few studies were available to assess publication bias or the effects of sex or species (Table S2).

## Discussion

This is the first meta-analysis that examines the effects of different rodent models of ELS on spatial learning providing several new insights to some key questions in the field. For example, we show that MS and LBN cause similar cognitive deficits in the MWM latency to find the platform, the probe trial, and the NOR test (Summarized in Fig. S2). The effect sizes of MS and LBN in these tasks were mostly moderate (Hedge’s *g* = −0.3–(−0.6), except for large effect size in the probe trial for LBN (Hedge’s *g* = −0.82). In contrast, exposure to handing showed improved performance in the latency to find platform and probe trial of the MWM, with no clear difference compared to control condition in the NOR (Fig. S2). These results demonstrate both quantitative and qualitative differences between handling and MS and LBN in the MWM and NOR and are consistent with previous work showing that handling causes different outcomes in stress reactivity^33,37^ and sensitivity to pain compared to MS^30^. Sustained elevation of CRH in the hippocampus has been shown to play a central role in inducing synaptic abnormalities and spatial-learning deficits in rodents exposed to LBN^12,38^, but its role in modifying hippocampal function in MS and handling has not been studied extensively. Nevertheless, Wang et al. found increased CRH levels in the hippocampus in rats exposed to MS, and administrating the CRH receptor antagonist (CP-154526) improved performance in the MWM and NOR in rats exposed to MS^39^. To the best of our knowledge, no group has yet shown that handling causes a reduction in CRH levels in the hippocampus, but work by Fenoglio et al. found that transient administration of CRH receptor 1 antagonist to control pups from P10-17, enhanced performance in the MWM and NOR to levels seen in handled animals. A similar procedure did not affect cognitive performance in handled animals, consistent with the notion that CRH levels are low in this group^40^. Additional studies are therefore warranted to compare CRH levels in the dorsal and ventral hippocampus (see below), across these three models of ELS.

One of the most intriguing findings of our analysis is that exposure to handling causes similar deficits in contextual freezing compared to LBN and MS (Fig. S2). This result needs to be interpreted with caution given the relatively small number of studies contributing to this outcome and that the impacts of handling and MS were only significant using fixed-effect analysis. Nevertheless, it demonstrates that handling shares some similarities with other ELS paradigms and raises the intriguing possibility that all three ELS paradigms cause similar deficits in the ventral hippocampus. This assertion is consistent with data showing that the ventral hippocampus plays an important role in contextual freezing^8,22–25^ and provides a parsimonious explanation for these seemingly conflicting results. Additional support comes from studies showing that the offspring of low licking and grooming (LG) dams show reduced long-term potentiation (LTP) and poor spatial learning in the dorsal hippocampus, but increased LTP in the ventral hippocampus^41^ and enhanced contextual freezing compared to offspring of high-LG^42^. Important differences in the effects of prenatal stress on the dorsal versus the ventral hippocampus have also been reported^43^. However, given the role that other brain regions, such as the prefrontal cortex, play in these tasks^17,18,26–28^, it is also possible that changes in these other areas and not the hippocampus are responsible for these behavioral outcomes.

The heterogeneity in LBN studies was significantly lower compared to both MS and handling studies across 3 of the 4 cognitive tests. Large heterogeneity was also found in a recent meta-analysis examining the effects of MS on anxiety-like behavior^31^. This is not surprising given the lack of standardization in the separation procedure associated with the MS and handling paradigms (e.g., number of days, length of separation, temp, single vs. whole litter separation). In addition, regular human contact in the MS and handling procedures may also increase variability due to the effects of the sex of the researcher and his/her experience scruffing and transferring animals^31^. Indeed, moderator analyses focusing on MS studies found that longer separation procedures (i.e., greater separation index) were associated with more severe cognitive outcomes in the NOR, but not MWM. Surprisingly, we found that incubating pups at higher temp during the separation procedure was associated with worse cognitive outcomes in the MWM (both latencies to find platform and probe trial) and were not able to examine this in the NOR because of the small number of studies available. This was an unexpected outcome because of the known sensitivity of rodent pups to hypothermia^44^ and will require additional replication.

A previous meta-analysis has found that maternal separation increases anxiety-like behavior in rats, but not mice^31^. This was not the case for hippocampal-dependent tasks where most of the outcomes were similar in rats and mice. The only exception was a performance in the latency to find a platform where rats exposed to LBN were significantly more affected than mice exposed to LBN (Fig. 1). A systematic review without meta-analysis conducted by Loi et al. reported that males had more cognitive deficits in non-stressful learning paradigms compared to female rodents^13^. This was confirmed for NOR in rodents exposed to MS, but overall there was no clear sex effect on hippocampal-dependent function. These different outcomes might be due to the inclusion of non-hippocampal learning tests in the Loi et al. study and the more quantitative approach used in this study.

## Conclusions

This work provides several new insights on the impact of different rodent models of ELS on spatial learning. First, LBN and MS, cause similar deficits in tasks such as the MWM and NOR that rely heavily on the dorsal hippocampus. In contrast, handling improved performance in the MWM and had no significant effect in the NOR. Second, all ELS paradigms, reduced contextual freezing, suggesting similar abnormalities in the ventral hippocampus and/or other brain regions. Third, heterogeneity was significantly lower in LBN compared to handling and MS. Fourth, ELS causes similar cognitive deficits in male and female rodents with no differences in the sensitivity between mice and rats.

## Supplementary information

Supplemental info
