# Supplementary material for: Deficits in hippocampal-dependent memory across different rodent models of early life stress: systematic review and meta-analysis
Source: Transl Psychiatry. 2021 Apr 20;11:231. doi: 10.1038/s41398-021-01352-4 (PMC8058062; doi:10.1038/s41398-021-01352-4)
Supplement: Supplementary file 1 — Supplemental info [file 41398_2021_1352_MOESM1_ESM.pdf]

Supplementary information

Methods

Figures S1-S6

Tables S1-S2

Methods

Selection criteria for included studies

The following criteria were used to determine eligibility for MS and handling: (1) Litters had to be randomized to either MS, handling or control condition. (2) Pups needed to be physically separated from their dams for 1-6 hours (MS), or less then 20min for handling, with separation taking place for 8-22 days beginning with the first 3 days of life [postnatal day (P) 1-3]. (3) During separation, pups needed to be removed from their home cage. They could be transferred to an incubator or kept at ambient temperature in a new cage. During the separation, pups could be kept as a group or individually isolated. (4) Dams could be removed or stay in the home cage, but could not be exposed to any additional stress during the separation period. (5) Nesting material was available to construct a nest. (6) Studies needed to have enough information to calculate effect size (number of animals per group, mean, Standard Error of the Mean or Standard Deviation). (7) Information regarding sex and age of testing needed to be available. (8) Testing for MWM, NOR, or CFC in offspring ages greater than P25 needed to be included. (9) Control groups needed to be raised under Animal Facility Rearing (AFR) or Non-Handled (NH) conditions. (10) Studies were conducted in rodents (mice or rats) and (11) were written in the English language.

Eligibility criteria for LBN included: (1) Litters were randomized to either LBN or control condition. (2) Pups needed to be exposed to LBN from P0-21, with or without mesh. (3) Pups could not be separated from the dam. (4) Studies needed to have enough information to calculate effect size. (5) Information regarding sex and age of testing needed to be available. (6) Tests for the MWM, NOR, or CFC in offspring ages greater than P25 needed to be included. (7) Studies were conducted in rodents (mice or rats) and (8) were available in the English language.

Data Extraction

For handling and MS, data collected from each study included author, year, species, strain, sex, age of testing, test (MWM, NOR, CFC), length of separation(hours/minutes), age of initiating the separation, duration of separation (days), temperature at separation, and single vs whole litter separation. For LBN, data collected included author, year, species, strain, sex, age of testing, test (MWM, NOR, CFC), test outcomes, mesh type, and age of starting LBN and duration (days). Test outcomes were collected as mean and variance measure (SEM and SD) by sex, and group (experimental and control). When data were only available in graphical form the program WebPlotDigitizer (Ankit Rohatgi, 2019) was used to extract numerical values using the distance measurement function [1, 2].

Behavioral measurements

Only few studies provided information on repeated measures ANOVA during training in the MWM and therefore the latency to find the platform during the last day of training was used to assess MWM performance during training (Fig 1). Date from the probe trial= percent time swimming in the correct target, was used to calculate Hedge's g for the probe trial (Fig 2). The preference for the novel object= time exploring the novel object/ time exploring both objects was used to calculate effect size for the NOR (Fig 3). Freezing time in response to context was used to calculate Hedge's g for the CFC (Fig 4).

Moderating effects of sex, species, separation index and separation temperature

The effects of sex and species (rats vs mice) was examined utilizing the Chi-square (X^2) test for subgroup differences [3]. Separation index was calculated for MS by multiplying the number of days pups were separated from the dam by the length of the separation in

hrs. Post-hoc, pairwise comparisons were conducted using the test for subgroup differences to compare outcomes across ELS paradigms when the initial test the Chi-square test for subgroup differences was statistically significant. A moderator analysis was used to assess the effects of separation index and temp of separation on cognitive performance in the three tests for MS using Comprehensive Meta-Analysis Version 3.0. All moderators were assessed individually after adjusting for species (and sex when necessary), the threshold of statistical significance was set at $p < 0.05$. Moderator analyses for separation index and separation temp were not conducted for handling because of the small number of studies available and for LBN because no maternal separation takes place during the LBN procedure.

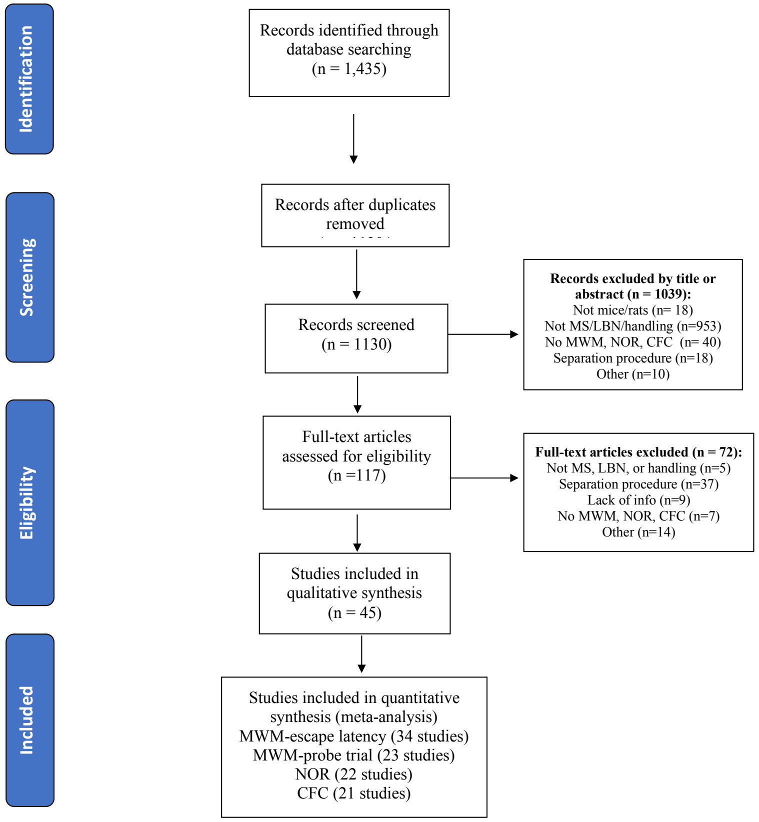

Figure S1. PRISMA flow diagram. A PRISMA flow diagram depicting the selection process and reasons for excluding studies from the analysis. Reasons for exclusion include studies not conducted in mice or rats (criteria 10), studies that did not use handling, MS, or LBN (criteria 1). Examples of studies that were excluded based on “separation procedure” include separation procedures for more than 6hrs daily or less than 8 days, not removing the litter from home cage, additional maternal stress, not providing nesting material, or using a split litter design (criteria 2-5). Studies that did not test for MWM, NOR, or CFC (criteria 8). Studies that were excluded under “other” category include studies that did not included appropriate control group or were not written in English (criteria 9 & 11). Studies that were removed because of “lack of information” included those that did not provide the number of animals or sex of the animals (criteria 6).

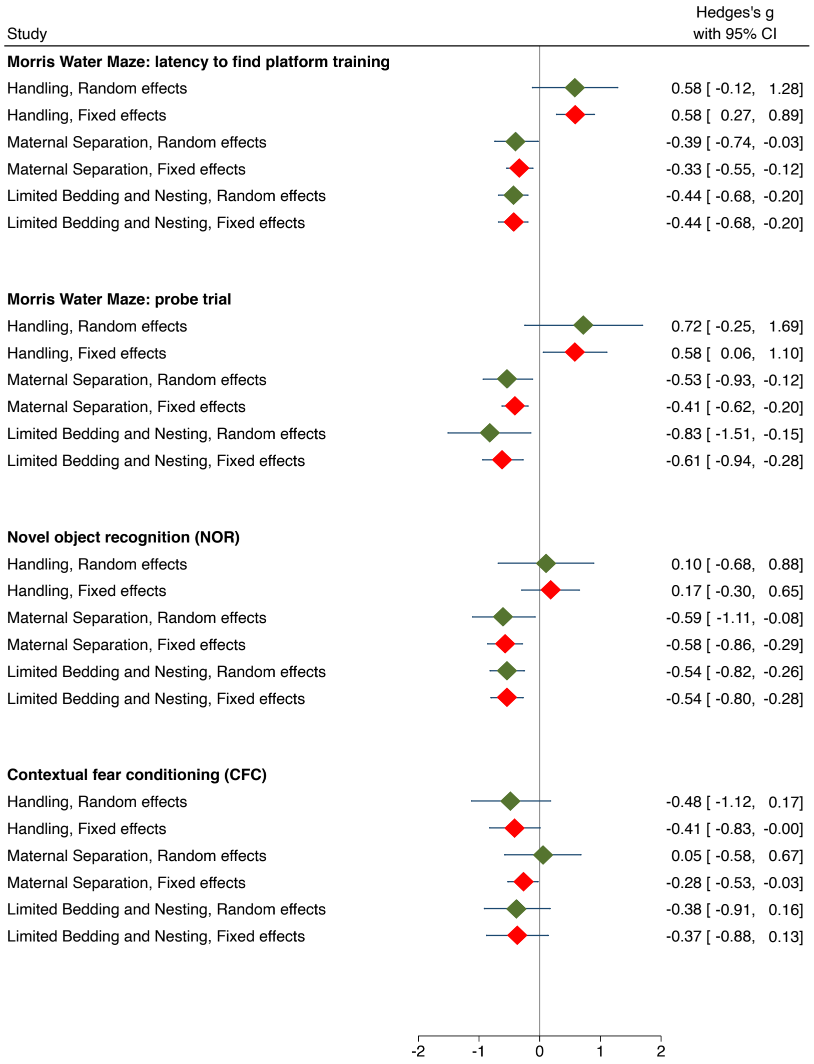

Figure S2. Forest plot summary of the effects of different rodent models of ELS on hippocampal dependent memory.

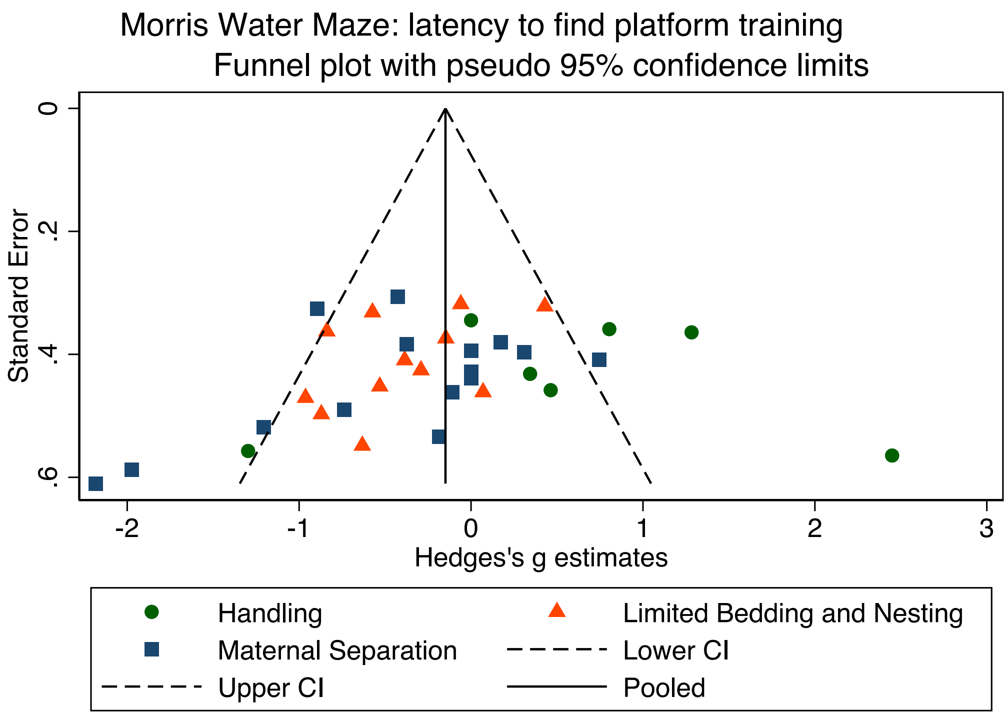

Figure S3. Funnel plots for studies looking at the effects of different rodent models of ELS on latency to find a platform in the MWM task.

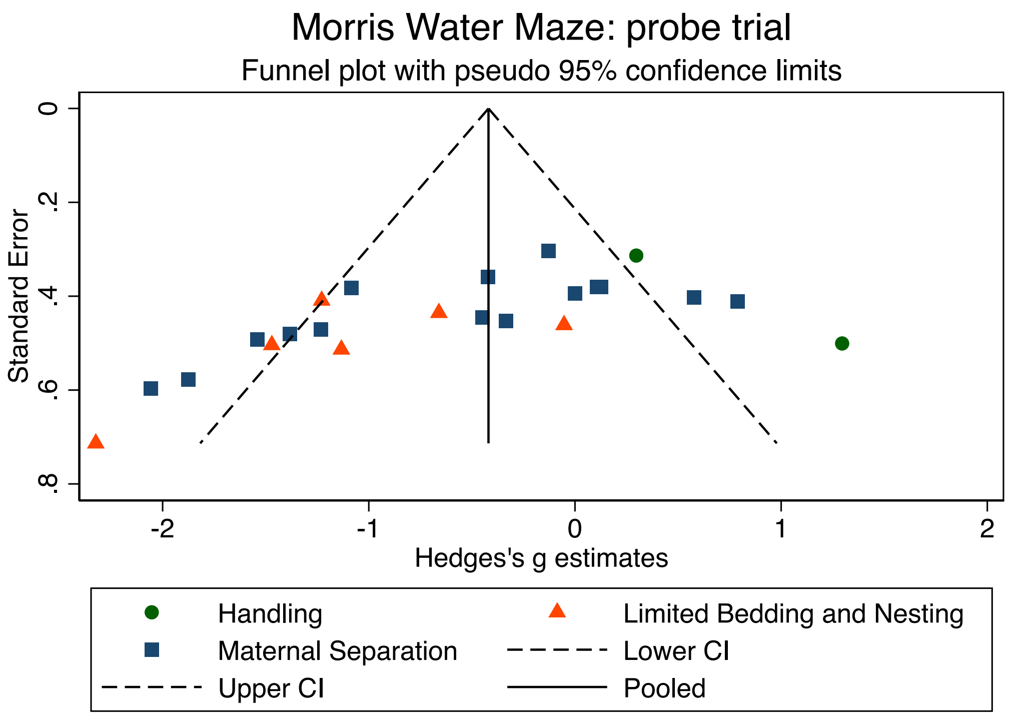

Figure S4. Funnel plots for studies looking at the effects of handling, MS, and LBN on performance in the MWM probe trial.

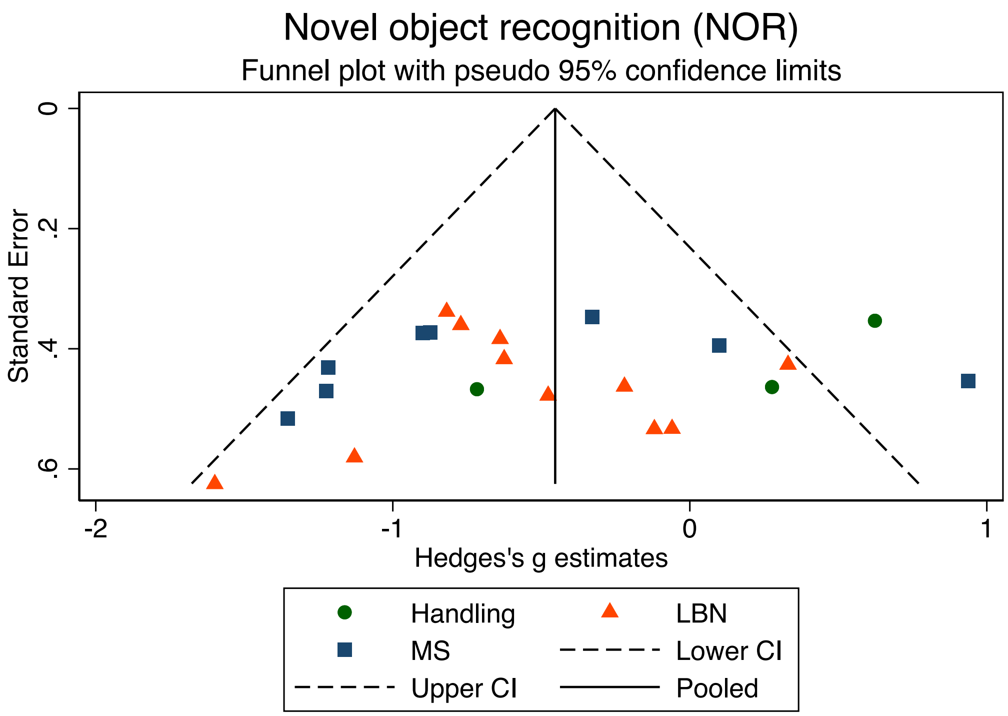

Figure S5. Funnel plots for studies looking at the effects of ELS on performance in the NOR test.

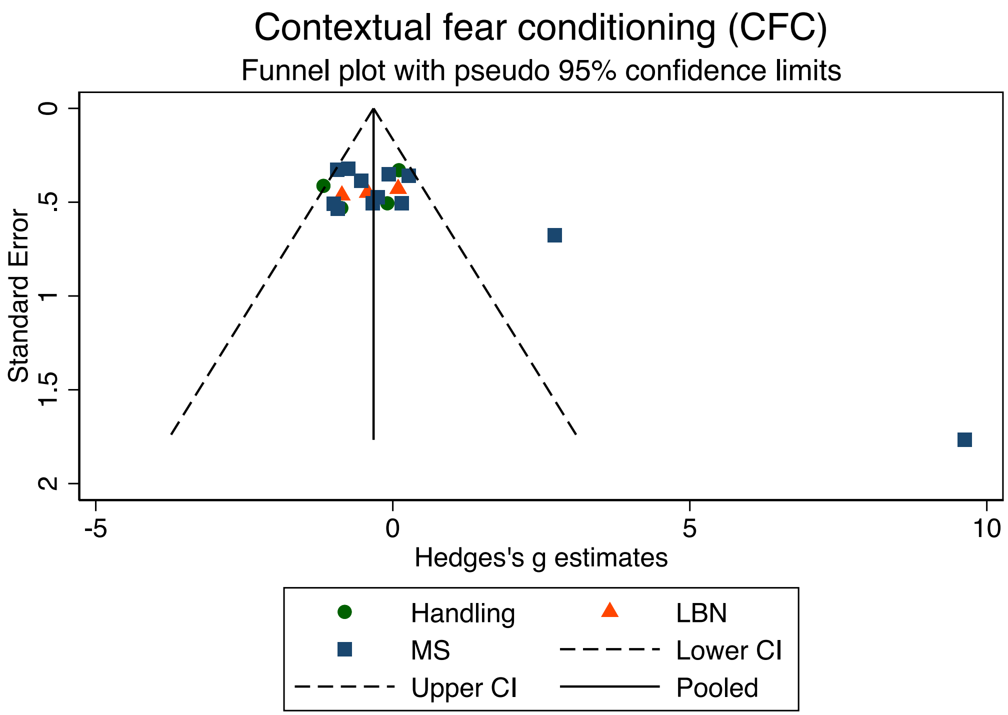

Figure S6. Funnel plots for studies looking at the effect of handling, MS, and LBN on freezing behavior in the CFC test.

Table S1. Detailed summary of all studies used in the meta-analysis. Abbreviations: Animal facility rearing (AFR), Females (F), Males (M), number of rodents per group (n), Non-handled (NH) Postnatal day (PND), Standard error of mean (SEM), Variance measured (VM). A link to the original excel file is available at: http://www.authorea.com/476416/1KAHz3kUglc11IvQpTt8Mg

Handling studies

Reference	PMID	Rat vs Mouse	Species-strain	Time handling	Days handled	Start handling (PND)	Separation Index	Room temperature (Celsius)	Incubator temp	Single vs whole litter during separation	Age of testing (PND)	Sex	Control Type (AFR, NH, EH)	test	test	figure	outcome	variance measure	mean	vm	n	mean	vm	n	mean	vm	n	mean	vm	n
Li, 2018	29678562	mice	BALB/cCrSlc	15	14	1	3.50	24	33	single	64	M	AFR	1	MWM	fig 3A	day (s)	SEM	34.81	8.6	8	23.84	6.66	10						
Li, 2018	29678562	mice	BALB/cCrSlc	15	14	1	3.50	24	33	single	65	M	AFR	2	MWM	fig 3B	quadrant	SEM	19.74	4.51	8	34.29	2.77	10						
Plescia, 2014	24216081	rat	wistar	15	20	2	5.00	22-28	30-32	single	55	F	AFR	3	NOR	fig 1	object	SEM							52.66	1.33	16	61.06	4.45	16
Plescia, 2014	24216081	rat	wistar	15	20	2	5.00	22-28	30-32	single	71	F	AFR	1	MWM	fig 3A	escape latency last day (s)	SEM							28.59	5.36	16	14.95	2.33	16
Plescia, 2014	24216081	rat	wistar	15	20	2	5.00	22-28	30-32	single	72	F	AFR	2	MWM	fig 4A	probe trial (s)	SEM							26.36	5.87	26	34.6	5.45	16
Guijarro, 2007	17697719	rat	wistar	15	13	2	3.25	23	32	whole	90	M	AFR	4	CFC	fig 1	percent freezing behavi	SEM	28.35	4.03	14	30.16	3.76	24						
Zaharia, 1996	8972542	mice	BALB/cByJ	18	21	1	6.30	22	22	whole	130	M	NH	1	MWM	1A	escape latency last day	SEM	41.21	4.3	17	22.19	2.49	18						
Zaharia, 1996	8972542	mice	C57BL/6ByJ	18	21	1	6.30	22	22	whole	130	M	NH	1	MWM	1A	escape latency last day	SEM	9.96	1.6	16	9.96	1.6	16						
Reshetnikov, 2018	no pmid	mice	c57B1/6 mice	15	13	2	3.25	not available	31	whole	90	M	NH	3	NOR	2B	with new object:total	SEM	0.61	0.06	8	0.51	0.03	10						
Fegnolio, 2005	15932935	rat	sprauge dawley	15	8	2	2.00	not available	not available	whole	60	M	NH	1	MWM	1B	escape latency last day	SEM	22.89	1.24	10	13.16	1.12	11						
Fegnolio, 2005	15932935	rat	sprauge dawley	15	8	2	2.00	not available	not available	whole	60	M	NH	3	NOR	3B	% perference of novel o	SEM	61	16	9	72	7.2	8						
Couto-Pereira, 2019	31191245	rat	wistar	10	10	1	1.67	22	32	whole	95	M	NH	4	CFC	1B	behavior	SEM	57.72	4.22	13	37.47	5.06	13						
Kosten, 2006	16626646	rat	sprauge dawley	15	22	1	5.50	not available	21	single	60	M and F	AFR	4	CFC	2A	behavior	SEM	79.93	7.39	8	57.4	11.62	6	77.11	10.22	8	73.95	14.97	6
Noschang, 2010	20369293	rat	wistar	10	11	1	1.83	22	31	whole	90	M and F	NH	1	MWM	1a M; 1b F	day (s)	SEM	30.94	6.05	10	24.39	5.49	10	17.93	2.83	7	32.57	4.89	7

MS studies

Reference	PMID	Rat vs Mouse	Species-strain	length of separation (1-6hr/d)	Start of Separation Period (PND)	Duration of Separation Period (7-22 Days)	Separation Index	Room Temperature (Celsius)	Incubator temp	Single vs whole litter during separation	Age of testing (PND)	Sex	Control Type (AFR, NH, EH)	test	test	figure	outcome	variance measure	mean	vm	n	mean	vm	n	mean	vm	n	mean	vm	n
banqueri, 2018	29908971	rat	wistar	4	1	21	84	22	30	whole	100	F	AFR	1	MWM	fig 4A	escape latency last day (s)	SEM							19.07	4.5	10	19.07	5.4	10
Xu, 2018	29759084	rat	Sprauge-Dawley	3	2	14	42	21	32	single	25	M and F	AFR	1	MWM	fig 8A, 3(m) a	escape latency last day (s)	SEM	14.32	4.28	8	23.01	3.55	8	12.13	4.69	8	24.72	4.37	8
Dalle, 2017	28549887	rat	Sprauge-Dawley	3	2	13	39	21	not available	whole	28	M	AFR	1	MWM	1A	escape latency last day (s)	SEM	7.01	1.85	20	36.55	10.06	20						
Dalle, 2017	28549887	rat	Sprauge-Dawley	3	2	13	39	21	not available	whole	58	M	AFR	2	MWM	1A	time spent in quadrant (s)	SEM	47.82	3.97	10	19.8	6.71	10						
Dalle, 2017	28549887	rat	Sprauge-Dawley	3	2	13	39	21	not available	whole	74	M	AFR	2	MWM	1A	time spent in quadrant (s)	SEM	34.6	2.74	10	15.8	5.14	10						
Zhang 2014	25157962	rat	Sprauge-Dawley	3	1	20	60	22	30	single	90	M	AFR	1	MWM	4B	escape latency last day (s)	SEM	12.26	1.91	12	8.18	1	12						
Zhang 2014	25157962	rat	Sprauge-Dawley	3	1	20	60	22	30	single	90	M	AFR	2	MWM	4C	percent time spent in quadrant	SEM	32.94	2.16	11	41.18	3.33	13						
Sun, 2014	24667363	rat	wistar	6	1	21	126	23	28	whole	90	M and F	AFR	4	CFC	2C	percent freezing behavior	SEM	95.24	4.35	7	87.3	10.85	7	88.09	10.07	9	60.58	19.31	7
Sun, 2014	24667363	rat	wistar	6	1	21	126	23	28	whole	94	M and F	AFR	2	MWM	3C	percent time spent in quadrant	SEM	29.08	10.46	13	32.65	6.89	13	26.28	12.76	13	30.61	10.97	13
Sun, 2014	24667363	rat	wistar	6	1	21	126	23	28	whole	95	M and F	AFR	1	MWM	3A	escape latency last day (s)	SEM	49.46	29.13	13	30.87	28.26	13	41.3	23.05	13	64.35	15.71	13
Chocyk, 2014	24508235	rat	wistar	3	1	14	42	not available	34	whole	35	M and F	AFR	4	CFC	2	percent freezing behvior	SEM	28.56	4.97	20	14.34	3.12	20	21.5	4.63	20	7.52	1.97	20
Baudin, 2012	22922490	rat	long-evans	3	1	14	42	22	32-34	single	83	M	AFR	1	MWM	3A	escape latency last day (s)	SEM	14.4	1.6	12	14.4	1.6	12						
Baudin, 2012	22922490	rat	long-evans	3	1	14	42	22	32-34	single	85	M	AFR	2	MWM	3B	percent time spent in quadrant	SEM	33.72	3.44	12	33.72	2.53	12						
Solas, 2010	20182419	rat	wistar	3	2	20	60	21	not available	whole	67	M	AFR	3	NOR	1B	percent preferece of novel object	SEM	77.98	2.38	15	58.93	7.14	15						
Solas, 2010	20182419	rat	wistar	3	2	20	60	21	not available	whole	547	M	AFR	3	NOR	1B	percent preferece of novel object	SEM	76.38	9.58	15	45.53	8.09	15						
Solas, 2010	20182419	rat	wistar	3	2	20	60	21	not available	whole	67	M	AFR	2	MWM	1A	quadrant(cm)	SEM	393.87	49.53	15	330.19	20.75	15						
Solas, 2010	20182419	rat	wistar	3	2	20	60	21	not available	whole	547	M	AFR	2	MWM	1A	quadrant(cm)	SEM	299.54	21.36	15	212.47	18.87	15						
Aisa, 2007	17307298	rat	wistar	3	2	21	63	not available	28-32	whole	105	M	AFR	2	MWM	5B	quadrant(cm)	SEM	425.8	34.93	10	310.98	19.35	10						
Aisa, 2007	17307298	rat	wistar	3	2	21	63	not available	28-32	whole	105	M	AFR	3	NOR	6B	%preference of novel object	SEM	72	4.28	12	54.54	3.69	12						
Guijarro, 2007	17697719	rat	wistar	3	2	13	39	23	32	whole	90	M	AFR	4	CFC	1	percent freezing behavior	SEM	28.35	4.03	14	26.98	5.15	17						
Uysal, 2005	16264401	rat	wistar	6	2	19	114	23	33	whole	31	M and F	AFR	1	MWM	1A	escape latency last day (s)	SEM	14.61	2.44	8	29.02	2.44	8	12.59	2.1	8	25.36	1.9	8
Uysal, 2005	16264401	rat	wistar	6	2	19	114	23	33	whole	32	M and F	AFR	2	MWM	1B	percent time spent in quadrant	SEM	36.25	0.92	8	28.13	1.83	8	37.67	1.58	8	29.04	2.75	8
Wang L, 2011	21331521	mice	BALB/cJ	3	2	14	42	not available	not available	whole	84	F	SFR	3	NOR	4B	%preference novel object zone	SEM							12.76	3.88	16	9.02	2.1	16
Zoicas, 2016	26497106	mice	CD1	3	1	14	42	22	30-33	whole	74	M	AFR	3	NOR	3B	%preference of novel object	SEM	59.3	5.71	12	61.33	5.71	12						
Asia, 2008	18554808	rat	wistar	3	2	20	60	21	not available	whole	68	F	AFR	3	NOR	4B	%preference of novel object	SEM							70.03	5.89	10	52.66	4.76	10
Couto-Pereira, 2019	31191245	rat	wistar	3	1	10	30	22	32	whole	95	M	NH	4	CFC	1B	percent freezing behavior	SEM	57.72	4.22	13	48.11	5.41	13						
Xiong, 2015	25576374	rat	Sprauge-Dawley	3	2	13	39	not available	not available	whole	70	M	AFR	4	CFC	2a	percent freezing behavior	SEM	56.06	4.39	14	60.91	4.56	16						
Xiong, 2015	25576374	rat	Sprauge-Dawley	3	2	13	39	not available	not available	whole	70	M	AFR	1	MWM	supp. Fig 1A	Escape latency last day (s)	SEM	12.82	3.65	12	9.59	1.88	12						
Xiong, 2015	25576374	rat	Sprauge-Dawley	3	2	13	39	not available	not available	whole	70	M	AFR	2	MWM	Supp fig 1B	time spent in quadrant (s)	SEM	67.12	5.94	12	79	5.5	12						
Xiong, 2014	24746487	rat	Sprauge-Dawley	3	2	13	39	not available	not available	whole	63	F	AFR	4	CFC	1B	%freezing	SEM							57.75	7.1	8	53.72	7.57	8
Cao, 2014	23712516	rat	Sprauge-Dawley	6	2	8	48	not available	32	whole	40	M	AFR	1	MWM	1A	escape latency last day (s)	SEM	14.09	12.86	10	14.09	12.86	9						
Cao, 2014	23712516	rat	Sprauge-Dawley	6	2	8	48	not available	32	whole	45	M	AFR	2	MWM	1B	time spent in quadrant (s)	SEM	53.12	3.83	10	45.92	6.08	9						
Reshetnikov, 2018	no pmid	mice	c57B1/6 mice	3	2	13	39	not available	31	whole	90	M	AFR	3	NOR	2B	object:total time)	SEM	0.61	0.06	8	0.42	0.03	9						
Pusceddu, 2015	25965872	rat	Sprauge-Dawley	3	2	11	33	21	32	whole	77	F	NH	3	NOR	2F	(familiar/total)	SEM							0.69	0.04	10	0.78	0.03	10
Lai, 2006	16316743	rat	Sprauge-Dawley	1	2	8	8	not available	30	single	50	M	non-isolated	1	MWM	3A	escape latency last day (s)	SEM	14.29	1.43	22	17.14	1.43	20						
Lai, 2006	16316743	rat	Sprauge-Dawley	1	2	8	8	not available	30	single	55	M	non-isolated	2	MWM	3B	percent time in target quadrant	SEM	39.32	2.18	22	38.02	2.18	20						
Huang 2002	12366727	rat	Sprauge-Dawley	1	2	8	8	not available	not available	single	84	M	non-isolated	1	MWM	2	escape latency last day (s)	SEM	15.4	4.03	8	16.44	2.04	9						
Kosten, 2006	16626646	rat	Sprauge-Dawley	3	1	22	66	not available	21	single	60	M and F	AFR	4	CFC	2A	%freezing	SEM	79.93	7.39	8	83.3	8.1	6	77.11	10.22	8	53.17	15.55	6
Diehl, 2014	24368626	rat	wistar	3	1	10	30	22	32	whole	60	M and F	NH	4	CFC	1 day 2	freezing(s)	SEM	210.26	52.74	8	867.15	100.83	8	153.04	30.66	8	937.27	65.84	8
Xue, 2013	24280707	rat	wistar	3	1	22	66	22	30	single	59	M	AFR	1	MWM	1a	escape latency last day (s)	SEM	34.64	2.98	6	36.46	4.31	6						
Diehl, 2012	22108759	rat	wistar	3	?	?	?	22	32	whole	70	M	seperated	2	MWM	2a	Time spent in quadrant (s)	SEM	20.38	2.08	9	18.54	1.32	9						

LBN studies

Reference	PMID	Rat vs Mouse	Species-strain	LB length(days)	Start of Stress Period (PND)	mesh	Age of testing (PND)	Sex	Control Type (AFR, NH, EH)	test	test	figure	outcome	variance measure	mean	vm	n	mean	vm	n	mean	vm	n	mean	vm	n
Hoeijmakers, 2018	29563870	mouse	C57B1/6J	7	2	stainless steel	273	m	AFR	1	MWM	Fig 3B	last day latency (s)	SEM	20.5	4.49	9	24.24	3.3	12						
Hoeijmakers, 2018	29563870	mouse	C57B1/6J	7	2	stainless steel	273	m	AFR	2	MWM	Fig 3c	% time spent in quadrant	SEM	28.86	3.01	9	23.18	2.21	12						
Hoeijmakers, 2018	29563870	mouse	C57B1/6J	7	2	stainless steel	60	m	AFR	3	NOR	1C	% preference novel object	SEM	65	5	9	73	8	12						
Cui, 2006	16790315	rat	sprauge dawley	20	2	wire	55	m	AFR	1	MWM	Fig 1a	last day latency (s)	SEM	19.31	0.5	8	28.07	4.73	8						
Cui, 2006	16790315	rat	sprauge dawley	20	2	wire	55	m	AFR	2	MWM	Fig 1b	time spent in target quadrant	SEM	71.43	6.8	8	49.79	5.94	8						
Wang XD Rammes, 2011	21940453	mouse	129S2/Sv X C57BL/6J	7	2	aluminium	180	m	AFR	1	MWM	Fig 1B	last day latency (s)	SEM	26.14	5.15	20	27.49	5	18						
Wang XD Rammes, 2011	21940453	mouse	129S2/Sv X C57BL/6J	7	2	aluminium	180	m	AFR	1	MWM	1B	time spent in target quadrant(s)	SEM	32.43	0.1	20	26.88	4.34	18						
Naninik, 2015	25269685	mouse	C57B1/6J	7	2	stainless steel	150	m and f	AFR	2	MWM	Fig 5G	percent time in quadrant	SEM	46.66	2.01	6	23.08	5.02	6	29.6	2.51	9	29.11	3.55	8
Naninik, 2015	25269685	mouse	C57B1/6J	7	2	stainless steel	150	m and f	AFR	1	MWM	Fig 5E males; 5F females	escape latency last day (s)	SEM	13.69	5.24	6	24	6.93	6	20.45	3.52	9	19.44	5.87	8
Naninik, 2015	25269685	mouse	C57B1/6J	7	2	stainless steel	150	m and f	AFR	3	NOR	Fig 1B	%preference of novel loccation	SEM	67	4.6	6	48	7.7	6	63	7	9	55	16	8
Burnson, 2005	16221841	rats	sprauge dawley	7	2	plastic	121-152	m	AFR	1	MWM	2a	escape latency (s)	SEM	7.61	1.28	8	9.95	1.38	11						
Burnson, 2005	16221841	rats	sprauge dawley	7	2	plastic	365	m	AFR	1	MWM	2b	escape latency (s)	SEM	7.47	1.95	8	26.95	7.47	11						
Burnson, 2005	16221841	rats	sprauge dawley	7	2	plastic	365	m	AFR	2	MWM	Fig 2c, data for 12M age	% time spent in quadrant	SEM	48.45	4.68	8	29.82	3.39	11						
Burnson, 2005	16221841	rats	sprauge dawley	7	2	plastic	365	m	AFR	3	NOR	Fig 2E, 12M	% preference novel object	SEM	66	7.2	9	50	6.8	15						
Kanatsou, 2017	28611594	mouse	C57Bl6	7	2	stainless steel	120	m	AFR	4	CFC	Fig 4 day 2	percent freezing behavior	SEM	11.93	2.11	10	12.68	2.87	10						
Kanatsou, 2017	27155103	mouse	C57BL/6N	7	4	wire	75	m and f	AFR	4	CFC	Fig 1C male and 1D fem	percent freezing behavior	SEM	76.16	4.15	11	70.08	4.83	8	74.18	8.1	7	56.96	4.87	14
Naninik, 2017	27770020	mouse	C5Bl/6J	7	2	aluminium	120	m	AFR	3	NOR	Fg 2A	% preference novel object	SEM	66	5	14	54	4.9	13						
Naninik, 2017	27770020	mouse	C5Bl/6J	7	2	aluminium	120	m	AFR	2	MWM	Fig 2E	%time spent in quadrant	SEM	46.31	2.79	14	30.08	4.15	13						
Naninik, 2017	27770020	mouse	C5Bl/6J	7	2	aluminium	120	m	AFR	1	MWM	2C-control, 2D-ES	escape latency (s)	SEM	16.98	5.71	14	20.06	5.3	13						
Rice, 2008	18566122	mouse	C57BL/6J	7	2	aluminium	120	m	AFR	1	MWM	fig 8a	escape latency (s)	SEM	18.05	3.83	10	23.91	4.51	13						
Rice, 2008	18566122	mouse	C57BL/6J	7	2	aluminium	243	m	AFR	3	NOR	fig 8b	%preferene novel object	SEM	61	3.9	6	52	5.7	13						
Ivy, 2010	20881118	rat	sprauge dawley	7	2	aluminium	334	m	AFR	3	NOR	Fig 3D	% preference novel object	SEM	68.6	6.6	20	50	2	13						
Ivy, 2010	20881118	rat	sprauge dawley	7	2	aluminium	334	m	AFR	3	NOR	Fig 5D	% preference novel object	SEM	72	6.6	23	50.5	2.9	15						
Ivy, 2010	20881118	rat	sprauge dawley	7	2	aluminium	334	m	AFR	1	MWM	Fig 3A	escape latency (s)	SEM	12.28	3.08	20	25.68	5.02	13						
Ivy, 2010	20881118	rat	sprauge dawley	7	2	aluminium	334	m	AFR	1	MWM	Fig 5A	escape latency (s)	SEM	13.6	3.08	23	23.77	5.37	15						
Molet, 2016	27657911	rat	sprauge dawley	7	2	aluminium	120	m	AFR	3	NOR	table 1	% preference novel object	SEM	65	9.7	6	67	15	6						
Molet, 2016	27657911	rat	sprauge dawley	7	2	aluminium	243	m	AFR	3	NOR	table 1	% preference novel object	SEM	65	6.2	6	62.7	8.2	6						
Molet, 2016	27657911	rat	sprauge dawley	7	2	aluminium	365	m	AFR	3	NOR	table 1	% preference novel object	SEM	69.2	6.2	6	48.4	3.1	6						
Manzano-Nieves, 2018	29781628	mouse	C57BL/6N	7	4	wire	75	m and f	AFR	4	CFC	Fig 1C male and 1D fem	percent freezing behavior	SEM	76.16	4.15	11	70.08	4.83	8	74.18	8.1	7	56.96	4.87	14

Table S2. Information about the number of studies, sex, and species for each behavioral test is summarized below.

**ELS Paradigm**	**Outcome Tested**	**Total number of studies**	**Number of studies in males(%)**	**Number of studies in rats(%)**
Handling	MWM- Escape Latency	7	5 (71%)	4 (57%)
MS	MWM Escape Latency	15	11 (73%)	15 (100%)
LBN	MWM Escape Latency	12	11 (92%)	5 (42%)
Handling	MWM -Probe Trial	2	1 (50%)	1 (50%)
MS	MWM Probe Trial	15	13 (87%)	15 (100%)
LBN	MWM Probe Trial	6	5 (83%)	2 (33%)
Handling	NOR	3	2 (67%)	2 (67%)
MS	NOR	8	5 (63%)	5 (63%)
LBN	NOR	11	10 (91%)	6 (55%)
Handling	CFC	4	3 (75%)	2 (50%)
MS	CFC	12	7 (58%)	12 (100%)
LBN	CFC	5	3 (60%)	0 (0%)

References

1. Morton RW *et al.* A systematic review, meta-analysis and meta-regression of the effect of protein supplementation on resistance training-induced gains in muscle mass and strength in healthy adults. *Br J Sports Med* 2018; **52**(6)**:** 376-384.

2. Wang D, Levine JLS, Avila-Quintero V, Bloch M, Kaffman A. Systematic review and meta-analysis: effects of maternal separation on anxiety-like behavior in rodents. *Translational psychiatry* 2020; **10**(1)**:** 174.

3. Israel H, Richter RR. A guide to understanding meta-analysis. *J Orthop Sports Phys Ther* 2011; **41**(7)**:** 496-504.
